# Supplementary material for: Whole embryonic detection of maternal microchimeric cells highlights significant differences in their numbers among individuals
Source: PLoS One. 2021 Dec 23;16(12):e0261357. doi: 10.1371/journal.pone.0261357 (PMC8699925; doi:10.1371/journal.pone.0261357)
Supplement: Attachment — Submitted filename: Response to Reviewers.docx [file pone.0261357.s001.docx]

Response to Reviewers

*5. Review Comments to the Author*

*Reviewer #1: The authors present a whole embryonic detection method for maternal microchimeric (MMc) cells using transgenic mice to test whether the number of maternal cells differs from one embryo to another. They justify their method of detection on the whole embryo instead of testing separated organs and tissues, as they hypothesize that if MMc does not differ in number from one embryo to another the overall number of MMc cells in the fetus may not differ among the different individuals.*

*And they further explain that different numbers of MMc cells have been reported among various tissues in previous studies and could reflect a particular accumulation in a specific tissue. They therefore want to overcome this by testing the entire embryo.*

*For this purpose, authors have crossed female mice heterozygous for the GFP locus (GFP+/-, from F1 BALB/c x C57BL/6-GFP) with non-GFP BALBc male mice, and offspring with no GFP gene (GFP-/-) were analyzed for GFP+/- MMc cells.*

*The major inconvenient with this system is that fetuses GFP-/- may have received microchimeric cells GFP+/- from other fetuses from the same litter. Therefore authors cannot assert that GFP-/+ cells are maternal.*

>Thank you for your constructive comment. We totally agree that we cannot fully exclude the possibility that GFP+ cells detected in embryos contain those of siblings in the same litter. We have thus made the following changes to our manuscript.

<Discussion> l. 262 ~ 272 in ‘Manuscript’

To add, another possibility was that the GFP+ cells identified here could be from GFP+/- siblings in the same liter, however, considering that the litter with the sample had relatively lower ratio of GFP+/- siblings compared to those of the other stages (the ratio of GFP+/- : GFP-/- embryos of the E18.5 was 0.23:1 in the sample litter, where it was 1:1.2 on average in the litters of the other stages. See also Table 1), and we also avoided using embryos with conjugated placenta, the probability of this sibling origin scenario appears to be less likely. Even a minor possibility would be that the embryo was a tetragametic chimera, leading to the high frequency of GFP+ cells, however, at least we could not find any sign of abnormality, including body size, morphology, and patchy signals of GFP. Thus, it would be fair to conclude that most of the cells detected here were MMc cells, or at least immunologically non-self cells.

In addition, we have added the following sentence according to the other comment.

<Discussion> l. 287 ~ 291 in ‘Manuscript’

Furthermore, while our experimental design cannot exclude the possibility of detecting GFP^+^ cells from GFP^+/-^ siblings in the same litter, this could be solved by crossing mother having GFP^+^/RFP^+^ on the same locus, with non fluorescent wild type male. In this design, GFP/RFP double positive cells in either GFP^+/-^ or RFP^+/-^ fetus should always be the cells of maternal origin.

Since the above idea is not our original, we have clarified that point in the acknowledgements section.

<Acknowledgement> l. 299 ~ 301 in ‘Manuscript’

We acknowledge one of the reviewers for providing a specific idea for discriminating maternal cells from cells of siblings.

*Moreover they observed that “MMc” differed in frequency among individual fetuses. The authors need to provide information about the other fetuses from the same litter. Were there more GFP + fetuses in the litter where one of them was very positive? How many fetuses from the same litter were analyzed? It is very difficult to discuss these results until we have this information.*

> We fully agree with the reviewer, and we have thus added the detailed information in the manuscript as Table 1. According to the table, the E18.5 embryo with the very high frequency of GFP^+^ cells had comparable, or even lower ratios of GFP^+/-^ siblings (average ratio of GFP^+/-^ : GFP^-/-^ was 1:1.2, while the sample litter at E18.5 was 0.23:1). We have added the following sentences in the discussion.

<Discussion> l. 262 ~ 272 in ‘Manuscript’

To add, another possibility was that the GFP+ cells identified here could be from GFP+/- siblings in the same liter, however, considering that the litter with the sample had relatively lower ratio of GFP+/- siblings compared to those of the other stages (the ratio of GFP+/- : GFP-/- embryos of the E18.5 was 0.23:1 in the sample litter, where it was 1:1.2 on average in the litters of the other stages. See also Table 1), and we also avoided using embryos with conjugated placenta, the probability of this sibling origin scenario appears to be less likely. Even a minor possibility would be that the embryo was a tetragametic chimera, leading to the high frequency of GFP+ cells, however, at least we could not find any sign of abnormality, including body size, morphology, and patchy signals of GFP. Thus, it would be fair to conclude that most of the cells detected here were MMc cells, or at least immunologically non-self cells.

*To absolutely demonstrate that maternal cells are maternal cells and not cells from other fetuses I would suggest to construct another model of MMc detection. Considering that this is technically feasible, one way out might be to realize the same experiences with mothers being heterozygous for Green and Red fluorescence, thus MMc would be GFP+/- RFP+/- among either heterozygous GFP+/- RFP -/- embryos or among heterozygous GFP-/- RFP +/- embryos.*

> We sincerely appreciate the proposal of this sharp experimental design. Although we do not have sufficient time to perform this additional experiment in the limited time under persisting COVID-19 situation, we have added this idea to the final discussion part of our manuscript as a future study. We also acknowledged the reviewer for this part to clarify his/her inspiring comment.

<Discussion> l. 287 ~ 291 in ‘Manuscript’

Furthermore, while our experimental design cannot exclude the possibility of detecting GFP^+^ cells from GFP^+/-^ siblings, this could be solved by crossing mother having GFP^+^/RFP^+^ on the same locus, with non fluorescent wild type male. In this design, GFP/RFP double positive cells in either GFP^+/-^ or RFP^+/-^ fetus should always be the cells of maternal origin.

> Since the above idea is not our original, we have clarified that point in the acknowledgements section.

<Acknowledgement> l. 299 ~ 301 in ‘Manuscript’

We acknowledge one of the reviewers for providing a specific idea for discriminating maternal cells from cells of siblings.

*Finally, a second important point: although this is a count of GFP + cells on a whole embryo, since the detection technique is done by FACS, it is a pity that other cell markers are not added to determine the phenotype. A multiparameter analysis would give additional indications as to the type of Mc cells. The study, nevertheless very interesting, must be reconsidered by the authors.*

> We thank the reviewer for this inspiring and constructive comment, as this is exactly what we originally wanted to perform. However, as there are overwhelming numbers of candidates to be detected by FACS we decided to give up on determining cell types. Instead, we planned to conduct the single cell RNA-seq to estimate the rough cell type repertoire based on their gene expression profiles in the near future, as described in the discussion section.

*Reviewer #2: In the manuscript “Whole embryonic detection of maternal microchimeric cells highlights significant differences in their numbers among individuals” the authors introduce a technique to examine the quantity of allogeneic cells in a murine model. Utilizing a breeding strategy to produce GFP- embryos in a GFP+ mother, the authors were able to utilize GFP+ cell presence in the embryos as an indicator of maternal microchimerism. To measure the quantity of GFP+ cells in the embryos they implemented a fluorescence-activated cell sorting strategy to filter cells on PI staining (for live cell detection) and GFP (for allogeneic cell detection). The presence of GFP+ cells, presumably from the mother, were identified in several of the embryos with one presenting with a notably larger proportion of GFP+ cells. This technique provides a useful tool for the analysis of maternal microchimerism in embryo development and may help to answer some of the enigmas of maternal microchimerism. This is an interesting report on a technique that can aid researchers in the understanding of fetomaternal chimerism and mechanisms in utero. I found the methods to be generally well described and took particular interest in the study design. I also have some comments for the authors to consider for further improvements in clarity and dissemination of their findings:*

*Major comments:*

*1. The sample size of the study is not clear from the text or figures. Samples sizes listed in lines 196-198 suggest a smaller number of samples compared to figure 3B. It would be helpful to clearly indicate how many litters and embryos were studied at each gestational age for the experiments.*

> We thank the reviewer for a constructive comment. To clarify this point, we have added the detailed information of sample sizes both for Figure 2B and Figure 3B, and further provided detailed information as an additional table (Table 1).

<Figure 3B legend> l. 239 ~ 242 in ‘Manuscript’

Sample numbers are as follows. control: E12.5: n = 5, E13.5: n = 5, E14.5: n = 5, E15.5: n = 5, E16.5: n = 5, E18.5: n = 4, sample: E12.5: n = 6, E13.5: n = 7, E14.5: n = 4, E15.5: n = 5, E16.5: n = 7, E18.5: n = 4]. See also Table 1 for more detail.

<Table 1> l. 246 in ‘Manuscript’

Please refer to the table we have newly added.

*2. The results section should include either a detailed section or table containing descriptive statistics for each gestational age group. As the study focus is in the prevalence and proportion of GFP cells at different gestational ages, I would suggest this to include data on GFP cell proportions, measure of variation, proportion of embryos with detectable GFP cells, sample size, etc.*

> We have added a new table (Table 1) to provide the detailed information.

*3. Line 91: The text states that “GFP+ embryos (identified by GFP excitation flashlight while embryos are in amnion) were carefully dissected from their sacrificial mother to avoid cross contamination of fetal and maternal cells.” As I read the rest of the study it refers to the study of the GFP- embryos for the presence of GFP+ cells. Is this a mistake or were the GFP+ embryos removed to avoid contamination of the GFP- embryos? If this is correct, perhaps this could be clarified by including in the schematic of Figure 1C.*

> Thank you for pointing it out. Yes. It was actually a mistake. We corrected it.

<Materials and Methods> l. 90 ~ 92 in ‘Manuscript’

To obtain suspensions of dissociated cells from the whole embryo, ~~GFP~~^~~+~~^GFP^-^ embryos (identified by the absence of fluorescence using GFP excitation flashlight while embryos are in amnion) were carefully dissected…

*4. Throughout the manuscript there is a continuous assumption that the source of the GFP+ cells is the mother, however there are other hypotheses described in the literature that could explain the source of microchimerism in utero. Assuming 50% of embryos in the litter are heterozygous (GFP+/-) it is difficult to determine the precise source of the cells. Anastomoses of placental vasculature has been previously demonstrated to produce twin-twin transfusion and could be another potential source of allogenic cells in utero. Were there any other measures used to confirm that the cells are of maternal origin? Is there any information or analyses that can be included about the heterozygous GFP+/- prevalence of each litter?*

> We totally agree with the comment that GPF+ cells could also be from GFP^+/-^ siblings in the same litter. Although our experimental design is not capable of discriminating cells from GFP^+/-^

mother or GFP^+/-^ siblings in the same litter, we have analyzed if the embryos with very high GFP+ cells had higher numbers of GFP^+/-^ siblings. The result suggested that the ratio of GFP^+/-^ siblings may not explain the high frequency of GFP^+^ cells. We have added the following sentences in the discussion.

<Discussion> l. 262 ~ 272 in ‘Manuscript’

To add, another possibility was that the GFP+ cells identified here could be from GFP+/- siblings in the same liter, however, considering that the litter with the sample had relatively lower ratio of GFP+/- siblings compared to those of the other stages (the ratio of GFP+/- : GFP-/- embryos of the E18.5 was 0.23:1 in the sample litter, where it was 1:1.2 on average in the litters of the other stages. See also Table 1), and we also avoided using embryos with conjugated placenta, the probability of this sibling origin scenario appears to be less likely. Even a minor possibility would be that the embryo was a tetragametic chimera, leading to the high frequency of GFP+ cells, however, at least we could not find any sign of abnormality, including body size, morphology, and patchy signals of GFP. Thus, it would be fair to conclude that most of the cells detected here were MMc cells, or at least immunologically non-self cells.

*5. Lines 214-217: Could you elaborate on the possible causes of the seemingly low number of GFP+ cells measured in the embryos? This could perhaps be added as part of a broader discussion of limitations.*

> We do not have definite answer to this, however, one possibility would be that the whole process took rather long time, and used FACS machine for soting. We have added this point in the discussion section.

<Discussion> l. 282 ~ 287 in ‘Manuscript’

Finally, major caveats of our methodology would be that 7 – 8 hours are needed to process embryos to obtain single celled suspension, and is not best suited to the mouse embryos from the latest stages. This, together with automatically removed cells in FACS as an electronic aborts (around 1 in 100 counts in our experiments) may explain the reason why we could not detect GFP^+^ cells in some of the embryos.

*6. Lines 249-255: It is stated that the large proportion of GFP cells identified in one sample is a product of the mother, but it should be considered that this may be a result of tetragametic chimerism (the fusion of two embryos in utero) or possible transfusion from another embryo.*

> That is a very important point, thank you. We have added the following sentence to our manuscript.

<Discussion> l. 262 ~ 272 in ‘Manuscript’

To add, another possibility was that the GFP^+^ cells identified here could be from GFP^+/-^ siblings in the same liter, however, considering that the litter with the sample had relatively lower ratio of GFP^+/-^ siblings compared to those of the other stages (the ratio of GFP^+/-^ : GFP^-/-^ embryos of the E18.5 was 0.23:1 in the sample litter, where it was 1:1.2 on average in the litters of the other stages. See also Table 1), and we also avoided using embryos with conjugated placenta, the probability of this sibling origin scenario appears to be less likely. Even a minor possibility would be that the embryo was a tetragametic chimera, leading to the high frequency of GFP^+^ cells, however, at least we could not find any sign of abnormality, including body size, morphology, and patchy signals of GFP. Thus, it would be fair to conclude that most of the cells detected here were MMc cells, or at least immunologically non-self cells.

*Minor comments*

*1. Lines 17-19: In the abstract it is stated that “maternal cells…migrate into the fetus and persist for the rest of their lives”, this appears to be an overstatement. While possible, there does not seem to be sufficient evidence to support this claim. It may be best to rephrase this into a phrase similar to “may persist for several decades” (see line 42).*

> Thank you. While maternal cells are detected in 89 years old patient with type one diabetes ([Chimerism.](https://www.ncbi.nlm.nih.gov/pmc/articles/PMC3023622/) 2010 Oct-Dec; 1(2): 45–50), we agree to tone down our statement to avoid overstatement.

<Abstract> l. 17 ~ 19 in ‘Manuscript’

During pregnancy in placental mammals, small numbers of maternal cells (maternal microchimeric cells, or MMc cells) migrate into the fetus and persist decades, or perhaps for the rest of their lives….

*2. Line 127: It is not clear if then entire material from each embryo was loaded onto the FACS machine or only a portion.*

> Roughly speaking, about one-fourth of total cell suspension volume was loaded onto the FACS machine. We have revised the sentence as follows.

<Materials and Methods> l. 126 ~ 129 in ‘Manuscript’

The cells (> 10,000,000 cells, which roughly corresponds to one-fourth of total cell suspension volume) obtained from a single fetus were stained with Propidium Iodide (PI) solution, and were loaded into a fluorescence-activated cell sorting (FACS) machine (BD AriaⅢu).

*3. In lines 246-249 it is stated that this study identified a comparable number of MMc cells of previous studies reviewed by Kinder et al. However, on review the review article by Kinder et al. does not appear to summarize maternal microchimerism levels (although it does for fetal microchimerism). In this case it would be better to cite the specific studies that produced comparable results.*

> Thank you for pointing this out. Actually, while some studies detected MMc cells using FACS in the organs with high MMc frequencies, no other studies analyzed frequency in the whole embryo. To be conservative, we have modified the corresponding part as follows.

<Discussion> l. 254 ~ 256 in ‘Manuscript’

Based on these results, we found that most of the embryos showed a comparable number of MMc cells, around 8 cells / 10^7^ sorted cells (Fig 3B). ~~which is consistent with previous studies reviewed in [4]~~.
