# Supplementary material for: Whole embryonic detection of maternal microchimeric cells highlights significant differences in their numbers among individuals
Source: PLoS One. 2021 Dec 23;16(12):e0261357. doi: 10.1371/journal.pone.0261357 (PMC8699925; doi:10.1371/journal.pone.0261357)
Supplement: Attachment — Submitted filename: Review Plos One september 27-21.docx [file pone.0261357.s002.docx]

**Comments to the authors**

The authors have carefully taken into account the comments of the two reviewers.

Table 1 was absolutely essential and allows a substantiated argumentation of data as to the probable maternal origin of these cells.

Moreover this table allows to note that when embryonic stages increase, “maternal” Mc is more abundant. This could be one of the reason why the embryo for which the number of "maternal" cells was the highest was the one tested at the latest embryonic stage.

Thus, although it is undeniable that there is an heterogeneity between individuals for the number of maternal microchimeric cells, since the embryos do not have the same number of Mc cells for the same embryonic stage; it seems there is also an effect due to the embryonic stage.

Indeed a Mann Whitney test allows to see that the number of maternal cells is statistically higher at E16.5 compared to all other previous embryonic stages (E12.5 + E13.5 +E14.5 + E15.5).

Thus it is very possible that later embryonic stages are those where MMc is the most perfused. Nevertheless a Spearman correlation does not show a statistical increase with embryonic stages (p=0.3), only a tendency.

The fact that there appears to be an effect due to the embryonic stage needs to be mentioned at least in the discussion and probably in the results.
