# Supplementary material for: Whole embryonic detection of maternal microchimeric cells highlights significant differences in their numbers among individuals
Source: PLoS One. 2021 Dec 23;16(12):e0261357. doi: 10.1371/journal.pone.0261357 (PMC8699925; doi:10.1371/journal.pone.0261357)
Supplement: Attachment — Submitted filename: Response_to_Reviewers.doc [file pone.0261357.s003.doc]

*Review Comments to the Author*

*Reviewer #1: please find details in the attached file*

*The authors have carefully taken into account the comments of the two reviewers.*

*Table 1 was absolutely essential and allows a substantiated argumentation of data as to the probable maternal origin of these cells.*

*Moreover this table allows to note that when embryonic stages increase, “maternal” Mc is more abundant. This could be one of the reason why the embryo for which the number of "maternal" cells was the highest was the one tested at the latest embryonic stage.*

*Thus, although it is undeniable that there is an heterogeneity between individuals for the number of maternal microchimeric cells, since the embryos do not have the same number of Mc cells for the same embryonic stage; it seems there is also an effect due to the embryonic stage.*

*Indeed a Mann Whitney test allows to see that the number of maternal cells is statistically higher at E16.5 compared to all other previous embryonic stages (E12.5 + E13.5 +E14.5 + E15.5).*

*Thus it is very possible that later embryonic stages are those where MMc is the most perfused. Nevertheless a Spearman correlation does not show a statistical increase with embryonic stages (p=0.3), only a tendency.*

*The fact that there appears to be an effect due to the embryonic stage needs to be mentioned at least in the discussion and probably in the results.*

> We sincerely appreciate your detailed investigation by further re-analyzing our raw data. Actually, we have performed similar analyses, but were a bit too conservative to refer in our manuscript. We have added this information to our manuscript.

Discussion Section l.272
Finally, considering that later stages are known to show higher frequency of MMc cells [23], it is intriguing that the sample with highly frequent MMc cells was detected for the latest embryonic stage. Consistently, while no statistically significant correlation was obtained between the number of MMc cells and developmental stages, however, we still found a weak tendency even without stage 18.5 (Spearman’s correlation coefficiency 0.3 for stages from 12.5 to 16.5).

*Reviewer #2: The manuscript “Whole embryonic detection of maternal microchimeric cells highlights significant differences in their numbers among individuals” details a technique for whole embryo analysis of maternal microchimerism. In a murine model the authors were able to utilize a breeding strategy of female mice heterozygous for fluorescent GFP with wild-type males to produce GFP- offspring. Using fluorescence-activated cell sorting, the authors were able to identify the presence of live GFP+ cells in some GFP- embryos, presumably originating from the mother. The study in several embryos found differences in GFP presence and quantity with one embryo presenting with a significantly larger proportion of GFP+ cells. The technique presented is well described and provides a useful approach for investigating the phenomenon of maternal microchimerism. The authors have appropriately addressed the reviewers’ comments and I am satisfied with the revisions made to address these concerns. In addition, I have a few additional comments that the authors should consider for the manuscript:*

> We sincerely appreciate your encouraging comments.

*1. The primary objective stated in line 67 indicates testing the frequency of maternal microchimerism among individual embryos during normal pregnancy. The authors make the conclusion that there is similarity in the quantity of GFP+ cells between the embryos however there appears to be notable variation in qualitative GFP+ cell detection. Now seeing the detail and distribution of results provided in Table 1, there should be some additional discussion regarding variation between litters. For example, litter D and litter H with 100% prevalence of GFP+ cell detection compared to other litters C and I at the same gestational ages.*

> This is actually a novel, important point that we overlooked. Thank you for the inspiring comment, and we have added the following sentence in our discussion part.

Discussion Section l.283
With this respect, it is tempting to know if the ratio of MMc^+^ detected embryos and those undetected differ among the litters (e.g., at least Litter C vs D, Litter H showed statistically significant ratio, see also Table 1).

*2. In the discussion section, lines 254-255, it is mentioned that “most of the embryos showed a comparable number of MMc cells, around 8 cells/ 10^7 sorted cells”. However, this estimation contradicts the data presented in Table 1 which (excluding the outlier) average 3.7 cells/10^7 sorted cells among samples with detectable GFP+ cells. Also, to improve clarity it would be helpful to indicate discussion of microchimerism results relating to “all embryos” or “GFP+ cell detected embryos”.*

> We totally agree with the comment, and we have made following modification to our manuscript.

Discussion Section l.254
Based on these results, we found that majority of the embryos showed a comparable number of MMc cells. Meanwhile, an unexpected finding was that one of the embryos at the latest stage (E18.5) showed 1,816 cells/10^7^ cells, which corresponds to a frequency approximately 500 times higher than the average of the other detected embryos (around 3.7 cells/ 10^7^ sorted cells for those detected with GFP^+^ cells but without the one with 1816 cells. See Fig 3B and Table 1).

*3. Further, in line 256-258 it is described that “one of the embryos at the latest stage (E18.5) showed 1,816 cells/10^7 cells, which corresponds to a frequency 1,000 times higher than that in the detected embryos”. This is true for some embryos with the lowest 1 cell/10^7 cells, but based on the average this would be approximately 500 times higher. Additional detail should be included in this sentence to help clarify the conclusions. This is also mentioned in the abstract line 27, and introduction line 71.*

> We have modified as follows.

Discussion Section l.254
Meanwhile, an unexpected finding was that one of the embryos at the latest stage (E18.5) showed 1,816 cells/10^7^ cells, which corresponds to a frequency approximately 500 times higher than that in the detected embryos.

In addition, we also corrected the corresponding parts in the abstract and introduction.

Abstract l.26
Using this technique, we found that the number of MMc cells was comparable in most of the analyzed embryos; however, around 500 times higher number of MMc cells was detected in one embryo at the latest stage.

Introduction l.69
We succeeded in developing an effective method for detecting whole embryonic MMc cells and found that the number of MMc cells was comparable in most of the analyzed embryos; however, around 500 times higher number of MMc cells was detected in one embryo at the latest stage.
